# Do we need a threshold conception of competence?

**DOI:** 10.1007/s11019-015-9646-5

**Published:** 2015-05-14

**Authors:** Govert den Hartogh

**Affiliations:** University of Amsterdam, Staten Bolwerk 16, 2011ML Haarlem, The Netherlands

**Keywords:** Competence, Threshold of competence, Decision-making ability, Decision-making authority, Variable standard of competence, Hard paternalism, Soft paternalism

## Abstract

On the standard view we assess a person’s competence by considering her relevant abilities without reference to the actual decision she is about to make. If she is deemed to satisfy certain threshold conditions of competence, it is still an open question whether her decision could ever be overruled on account of its harmful consequences for her (‘hard paternalism’). In practice, however, one normally uses a variable, risk dependent conception of competence, which really means that in considering whether or not to respect a person’s decision-making authority we weigh her decision on several relevant dimensions at the same time: its harmful consequences, its importance in terms of the person’s own relevant values, the infringement of her autonomy involved in overruling it, and her decision-making abilities. I argue that we should openly recognize the multi-dimensional nature of this judgment. This implies rejecting both the threshold conception of competence and the categorical distinction between hard and soft paternalism.

## An unsolved issue regarding competence

Decision making capacity or competence is the ability, or rather the set of abilities, needed for making decisions regarding one’s own life. There is a more or less general consensus about the abilities which we should reckon to belong to the set: the ability to distinguish between the alternatives at hand, to understand the major relevant consequences of each of those choices, to take the probability of their occurrence into account in some rough-and-ready way, to evaluate those consequences in view of one’s own values, and to actually make the decision recommended by this evaluation.^1^ People have all these cognitive, but perhaps partly also affective abilities, to a greater or lesser degree. Competence is therefore a concept that allows for degrees; the level of someone’s competence could be marked on a scale (Presuming that the relevant abilities are roughly commensurable with each other).

In practice, however, the first and foremost question medical, legal and other professionals usually ask is whether someone is competent or incompetent. The concept of competence then acquires a binary character. Apparently a certain threshold on the scale in question is involved.^2^ Many discussions of competence are flawed from the outset because they do not clearly distinguish between the scalar and the binary sense of the term.^3^ It is sometimes claimed, for example, that a person is empowered to make a decision for herself, if she is able to understand the information relevant to making it, and to weigh that information in the process of making her decision, but it is not specified how much understanding and appreciation are needed.^4^ If no imperfection of understanding or appreciation would be allowed at all, few people would ever correctly be judged competent to make any decision. If the scalar and the binary sense of competence are insufficiently distinguished, it will often also be unclear whether an assessment of competence makes a descriptive or a normative claim. If we ask to what extent a person has a certain cognitive ability, there is a fact of the matter to be tracked by the answer, but it is a normative judgment that this level of ability is enough for allowing the decision the person actually makes to stand, whether or not it is the right decision for her to make.

There is, secondly, also consensus about the fact that competence in this binary sense is a task-related concept: people may be competent to take decisions of a certain type, such as a decision about their diet, and incompetent to take decisions of another type, such as decisions about the management of their estate (Buchanan and Brock 1990, 18–23; Culver and Gert 1990, 619ff). Health care professionals do not always do justice to this task-related nature of the concept: patients are often classified as competent or incompetent *tout court*. It is, of course, possible to be categorically incompetent; think of babies and patients in a coma. You can hardly be competent to make a certain decision, if you are unable to make any decision at all. But where, among others, patients with Alzheimer or psychiatric patients are concerned, it is of major importance to focus the question about their competence on a certain decision or a certain category of decisions, for example to consent to a certain kind of proposed medical treatment or to refuse it.^5^

A third point of consensus concerns the presumption of competence. We assume in the case of most patients, and of most persons generally, that they are competent in all their decisions until a reason for doubt arises, we do not investigate their competence in the case of each and every decision. That would be impossible in practice, but there is a more fundamental reason for the presumption as well: we can only take each other seriously and treat each other as moral equals on this basis.

The presumption of competence in the context of health care often means that a patient who agrees to the proposal of treatment that his doctor regards as the most suitable is considered to be competent, whereas a patient who is in the same situation but refuses the treatment is solely on that ground subjected to investigation of his competence. This asymmetry is sometimes called into question for already harbouring an element of paternalism: we only raise problems with regard to people who deviate from the standard (Ganzini et al. 2005). However, the alternative would be to let go of the presumption of competence itself, and examine patients’ competence in all cases, whether or not there is any reason for doubt. That would really be paternalistic in the extreme.

A final point of at least apparent present consensus should be mentioned. Whether a person is sufficiently competent to make a certain decision, it is hold, should always be decided upon without reference to the (token-)decision she actually makes. This claim, however, can be made in a weaker and a stronger form. According to the weaker form one should never assess an unwise decision as such as having been made incompetently, one should always take the reasons for making it into account.^6^ According to the stronger form one should abstract from the token-decision altogether, and only consider whether the agent has the general abilities for making decisions of that kind (E.g. Culver and Gert 1990).^7^ It is only the weaker claim which I will assume to be true. For why should it be decisive that you have sufficient abilities for fulfilling a task of a certain standard type, if the particular task you have to execute differs from others of that type in precisely the one respect, or the particular configuration of relevant aspects, which explains your present failure (Wijsbek 2000)?^8^

Against the background of these four points of consensus (Buchanan and Brock 1990, ch1; Berghmans 2001; Charland 2008) the issues may be distinguished which are still controversial. Some of these I have flagged already in passing. It is often suggested that the most important element in the set of abilities comprising competence, indeed the ability which encompasses all others, is the ability to deliberate. However, one can very well ask an agent for his reasons without presupposing that he has been aware of them before he acted. Even spontaneous choices, triggered by emotions, normally can be explained by reasons.^9^ An additional relevant capacity is the ability to correct oneself: if the spontaneous emotional response leads to a choice which from the point of view of the agent’s priorities is a problematical one, a red light should come on which enables him to adapt his choice. But this process of adaptation, too, need not be gone through consciously. In the final analysis, deliberating ability may still be essential for fine-tuning one’s corrections in complex environments. But it is less central to competence than has often been supposed.

A second controversial issue concerns the role of emotions in decision-making. Emotions are needed for short-circuiting deliberation in relatively simple or recurring contexts, and for not only recognizing what should be done but also resolving to do it.^10^ “To decide is to jump, and in order to jump one needs emotions” (Wijsbek 2000, 85). The question in dispute is whether or not a judgment of competence requires us to appraise these emotions. True, it is often difficult to judge whether a surprising emotional response to a situation is nevertheless an appropriate one, given the agent’s beliefs and priorities, and if we allow such evaluations of ‘affective ability’ to be relevant we may risk losing the reproducibility of assessments of competence across assessors (Grisso and Appelbaum 2006 commenting on Tan et al. 2006). But that is not a good reason for denying these evaluations to be relevant, if they are.

A third major issue of controversy, which will turn out to be relevant for my argument at a later stage (“Assessing the theories” section), is whether in assessing the quality of a person’s decision we should always start from her present values and goals (which we often may infer from her emotional responses) or should consider to what extent these present priority ranking fits a pattern of more permanent values underlying her actions during at least a certain period of her life.

The fourth controversial issue is the one that I want to focus on. It concerns the proposal to use a variable standard when judging for competence in the binary sense.^11^ More specifically, on this view the answer to the question whether we regard a patient as sufficiently competent to make a certain decision should depend on what is at stake in making that decision. The point where we draw the line on the scale of competence to mark competence in the bivalent sense, must be placed higher when the risks involved in the decision (which the patient considers to make) are greater: when the damage that may occur to him as a result of that choice is more severe, or the chance that it will occur is higher. By setting stricter requirements we decrease the chance that a patient makes a decision by which he seriously harms himself. The alternative view, sticking to a non-variable standard, is that this risk, minor or major, is irrelevant to determinations of competence. The question whether we may regard the individual as sufficiently competent to make a certain decision then solely depends on his cognitive and affective abilities. If we also take risks into consideration, we would in fact, according to this view, while pretending to merely assess competence, introduce a paternalistic policy that protects people against the harmful consequences of their own decisions (Cf. footnote 44).

In the following sections I will first of all (“The symmetry argument” section) discuss a major argument against the introduction of a variable standard for competence in the bivalent sense. That discussion should help us to identify the basic issue underlying this particular dispute. The issue will appear to concern the question whether or not the authority to make a certain decision should be taken to depend only on considerations of competence in the scalar sense, i.e. on the relevant cognitive and affective abilities. Next I will distinguish the various answers which can be given to this question (“The distinction between hard and soft” section). Finally, I will discuss which of these answers we should prefer (“Assessing the theories” section). My conclusion will be that the normative view that is expressed in the proposal for a variable standard (the view that the attribution of decision-making authority should depend on other considerations besides competence in the scalar sense) is to be preferred, but that that view is expressed in a misleading way in the proposal itself. The accusation that it tends to conceal paternalistic considerations is well-founded. As soon as we understand the real import of the underlying normative view, we are driven to far more revisionistic conclusions as regards the orthodox view of people’s normative authority and the justification of paternalism than supporters of the variable standard usually recognize. That orthodox view is that ‘soft’ paternalism is sometimes justifiable, but ‘hard’ paternalism never is. Our discussion of the variable standard will lead us to reconsider this very distinction and its moral importance.

## The symmetry argument

Perhaps the most interesting argument brought forward against the variable standard of competence is the so-called symmetry argument, which has most systematically been developed by Mark Wicclair (1991, cf. Wicclair 1993, 11–20).^12^ He points out that the variable standard has as a result that a person who is faced with two options—for example to accept or reject a proposal of medical treatment—may be seen as competent when she chooses one option and as incompetent when she prefers the other, if the last option is much riskier than the first (Acknowledged by Buchanan and Brock 1990, 51–52). According to Wicclair this lack of symmetry is unacceptable where judgments on competence, and abilities in general, are concerned.

Is this true? If we focus on abilities in the scalar sense, the opposite would sooner be true. If we ask someone to make a certain calculation, and he comes up with the wrong answer, this is at least an indication that he is not able to make the calculation. Because ‘ability’ is a dispositional term, it is an indication only, not a proof, but our suspicion may grow into something close to certainty, when he tries again in all sorts of circumstances and keeps failing. However, if he answers correctly straightaway, we reasonably trust that he has the relevant ability. The same goes for decision-making competence: in any case in which the right decision can be clearly identified, the person who takes that decision can apparently face such tasks, but whoever makes mistakes raises doubts about his ability.^13^

How does the symmetry-argument fare when competence in the bivalent sense is concerned? In order to answer that question we have to understand why we want to identify a threshold of ‘sufficient’ ability to begin with. As I already suggested in the “An unsolved issue regarding competence” section, what we really want to know is whether the agent has the degree of competence that is sufficient to attribute to her the power to make a certain decision. If she has that authority, her decision will count, whatever it is. The essential characteristic of the concept of ‘authority’ is content-independence.^14^ When we impute epistemic authority to our doctor, we believe him when he says that a medicine has certain side-effects, and we also believe him when he denies that. When we assign legislative authority to our government, we accept that we have to drive on that side of the road, right or left, which it stipulates. When a patient has a right to ‘informed consent’, it is up to him, once he has been adequately informed, whether the treatment proposed can go on or not. The advice, the statute and the patient’s decision constitute reasons to believe something or to act in a certain way, but the reasons do not depend on what is advised, prescribed or consented to, only on the fact that it is. That is why these speech acts have authority. Their authority is only fully recognized when these reasons are accepted as decisive ones, although less than full recognition is not impossible.

It is for this reason that a judgment of competence in the bivalent sense cannot be simply overruled by pointing out that the patient made an unwise decision. If the decision you make is generally acknowledged to be the correct one, you need no claim to authority to have it respected, but only when your authority is recognized, your mistaken decision (or your correct decision believed to be mistaken) stands equally. In law the very terms of competence and capacity are often used to designate authority, rather than the set of mental capacities required for assigning it. But that is a confusing usage, particularly unfortunate because it amounts to an unnecessary use of legal fictions.

Because authority is content-independent in this sense, the corresponding judgement of binary competence is content-independent as well. Hence Wicclair’s point is basically correct. But we should be clear that the symmetry argument fundamentally refers to the authority to reach decisions, and to competence only in as far as the threshold of the relevant capacities is concerned deemed necessary to attribute this authority. If a person is not competent enough to accept a proposal, she is not competent enough to reject it either, because what she basically is not competent enough to do is to make the choice authoritatively. Hence, if we reach the conclusion that a patient is incompetent when he refuses treatment, this implies that his acceptance, too, would have been invalid, and that we, in case he should change his mind, still need the consent of a legal representative.^15^ Usually, this implication is obscured by the presumption of competence. It may be that if the patient consents there is no reason to investigate his competence, but if he refuses, there is. However, if the conclusion following from that investigation is negative, it holds for the consent as well as for the refusal.

Wicclair is right, then, but his point is a more limited one than he recognizes. It does rule out some versions of the variable standard, but not all of them. Suppose we only investigate a certain patient’s competence, because faced with a choice between A and not-A, he is inclined to choose A which might damage him. Our eventual conclusion cannot be that he is incompetent to choose A, but is or would be competent to choose not-A. He is incompetent to make a choice between A and not-A due to the risks involved in one of the options; it does not matter whether this is the option he is in fact inclined to choose. Nevertheless, the risks seem to be a relevant element in the characterization of the task that this patient faces. The task is always to choose between options. Because a judgement of (bivalent) competence is relative to the task in question, a higher degree of competence could still be required in the event of a task with greater risks. The symmetry argument does not at all exclude that possibility. It only excludes splitting up the authority to make a certain choice into the authority to reject an option and the authority to accept it.^16^

The limited force of the argument can be explained as follows. The power to authoritatively make a decision must to some extent be independent of the contents of that decision, but the extent need not (or maybe even cannot^17^) be unlimited. A medical practitioner may put forward all sorts of proposals for treatment that I cannot assess myself, and therefore I wisely rely on his epistemic authority when he proposes that I take medicine A and not B. But if he says that I should be wary of earth rays, I do not take that advice seriously, and may even start wondering whether I should not stop relying on his advice altogether. The sergeant may order the soldier to move forward when attacked, but he cannot order him to empty his pockets (Locke (1690) 1960, § 139).^18^ In the same way, a patient’s authority to decide for himself may only be independent of contents within certain limits. He may choose from among options A, B and C, even though option A would clearly be his best choice, and his ability to assess his own interests is less than optimal. But if he may choose between A, B, and C, it does not follow that he also may choose between A, B, C and D, if D is even more damaging to his interests than B or C.^19^ Perhaps we should allow him the choice, perhaps we shouldn’t: this cannot be decided on the basis of the symmetry argument. That argument, therefore, does not refute the proposal to use a variable standard of competence.

## The distinction between hard and soft

In order to identify the possible positions one could take as regards that proposal, it will be helpful to consider the distinction between hard and soft paternalism and it’s moral importance. The distinction has been made in the literature for quite some time now.^20^ The paradigmatic case of paternalism occurs when you act contrary to a person’s wishes in order to prevent her well-being being affected adversely. In such cases you do not recognize the person’s authority about the choices to be made for her own good, at least not as a decisive consideration. It is possible for you, however, not to recognize that authority, even when you comply with her wishes, for example when your decisive reason is that frustrating those wishes would by itself be a setback to her interests. That should also be considered a case of paternalism (Groll 2012). Paternalism is hard when the person is deemed competent, soft when she is not.

The distinction is often illustrated by an example of John Stuart Mill: if you know that a bridge across a canyon is about to collapse, that is a very good reason to stop someone who is about to cross that bridge, and make sure that he is aware of the danger (Mill 1986, 111). Joël Feinberg remarks, on the basis of this example, that soft paternalism is in fact no paternalism at all (Feinberg 1986, 12–16). The reason is that you only stop the person because you assume, until the opposite is proven, that he does not ‘really’ want to endanger his life, and that he is merely unaware of the danger. You therefore only act against his actual wishes in order to make room for these real wishes, just as you do when you act against his actual wishes if these are the product of fraud. The objections that can be made against hard paternalism therefore simply do not apply to the soft variant.

But this analysis cannot be extended to all cases of soft paternalism. In Mill’s example, you may expect that the person in question will be happy once the reason for the intervention has been explained to him. Should he have been annoyed about it in the first place, that annoyance will quickly evaporate. For he has no particular investments in his belief that the bridge is safe and will be quite willing to change his mind on being presented with evidence to the contrary. However, in many instances of soft paternalism this is not the case, e.g. when a demented patient is denied the authority to cook her own meal or to spend her own money. She will rightly feel that her wishes are not respected at all, she will feel frustrated in her desire to be her own person and she will feel that she is not recognized as an equal among equals.^21^ Maybe we can still in some sense maintain that the choice is not truly hers (see “Assessing the theories” section), but we cannot expect her to agree to this view if we explain it to her. In that case we can justify our interference with her liberty, but not *to her*. That we want to avoid that position is one of the reasons why we attribute to them the authority to take decisions regarding their own life to begin with, irrespective of whether these decisions are right or wrong. The same reason may plead against soft paternalism as much as against hard paternalism.

This is not the only reason to avoid soft paternalism, wherever possible. It is desirable to prevent unnecessary conflicts, in the case of someone who is deemed to be incompetent as much as in that of a supposedly competent person. It keeps intact a relationship of mutual trust, for example between doctor and patient. Medical action is often likely to be more successful when a patient heartily collaborates. But the most important reason is still that soft paternalism, too, often means an infringement of a person’s self-esteem. For such reasons it is normally important to obtain a person’s consent, whether she is considered to be competent or not.

Soft paternalism is not morally unproblematic, generally speaking. How should we judge hard paternalism? The position taken by Mill and Feinberg is that it should always be avoided.^22^ It seems that the proponent of the variable standard is also of this opinion, but in fact to him it is merely a tautological truth.^23^ It is clear that he recognizes two relevant considerations for the attribution of the authority to make a certain choice: the degree of scalar competence and the level of risk involved in making that choice. A greater competence may make up for higher risks. As soon as someone is considered sufficiently competent to decide, his decision carries authority. The question whether hard paternalism may nonetheless be justified in such a case cannot arise any more: all possible grounds for justification have already been taken into account in the process of attributing competence in the binary sense.

That, at least, is true if no other considerations can be relevant apart from scalar competence and risks. Are these really the only ones that the proponent of a variable standard could possibly take into account? In the debate about the possible justification of hard paternalism two such further considerations are mentioned regularly. First of all, the degree to which someone’s choice may express values which are characteristic for the way he leads his life. Are there issues at stake with which he is greatly concerned? In some cases it is hardly thinkable that someone makes a certain choice for other reasons than a lack of elementary prudence. In those cases there are few costs involved in denying him a choice or making it difficult for him to make it. It is somewhat extravagant to claim that the obligation to wear safety belts or moped helmets is detrimental to your self-esteem.^24^ A second relevant consideration could be the nature of the infringement of someone’s freedom. The obligation to carry a moped helmet or the prohibition to have a total body scan is much less of an infringement than being locked up in an institution, or suffering forced medication or nutrition or sterilization (Gezondheidsraad 2008, 102–104). In the following I will focus on risks, but I do not wish to dispute the relevance of these additional considerations for the attribution of decision-making authority.

There are authors who claim to reject the variable standard, but accept hard paternalism to prevent possible injury in some cases^25^ (E.g. Culver and Gert 1990).^26^ The resulting position differs only verbally from the variable standard: when we ask these authors and the proponents of the variable standard what conditions must be fulfilled if someone’s decision is to carry authority, their answer is exactly the same. The only difference is that these authors arrive at that answer by considering decision-making abilities and other relevant considerations, in particular risks, consecutively rather than simultaneously. It does then become unclear, however, what the result of the first step of this two-step procedure amounts to. ‘The person involved possesses a sufficient degree of competence.’ Sufficient for what? Apparently not for attributing authority to her since this depends on still other elements.

The only position which is substantially different from the variable standard as well as prima facie plausible is the position taken by Mill and Feinberg. According to that view the attribution of ‘sufficient’ decision-making competence solely depends on the possession of the relevant cognitive and affective abilities, all other considerations are irrelevant for attributing authority. That authority can therefore not be limited at a later stage by those other considerations. The result is an absolute prohibition of hard paternalism.

All in all, there are only two conceptions of the proper conditions for recognizing someone’s right to self-governance in a particular case worth considering. According to the first view the recognition of this right is solely dependent on competence in the scalar sense, and beyond the threshold paternalism is strictly forbidden. The other view weighs in elements from different dimensions, and a deficiency in one dimension (for example risk) may be compensated by a surplus in another dimension (for example scalar competence). The result is a variable standard of (binary) competence. I will refer to the first conception as the one-dimensional and to the second conception as the multi-dimensional theory of decision-making authority.

## Assessing the theories

Which theory should we prefer? The multi-dimensional theory recognizes a plurality of values which have to be taken into account, and sometimes weighed against each other, in particular the value of personal autonomy and of well-being. In the one-dimensional theory, to the contrary, it is only the value of autonomy that counts. That value is given a lexical priority to all other values. This theory may therefore be criticized by objecting to that ranking. No doubt it is of eminent importance to people to be given the opportunity to shape their own lives, and to be recognized as having the sovereignty to do so, but why should these interests always carry more weight than other interests, such as the interest in having a life at all which may be shaped in one way or another? (Cf. Arneson 2005 on Feinberg)^27^ Such objections belong to external criticism, criticism inspired by a rival value theory. But I want to claim that the one-dimensional theory can also be subjected to internal criticism, based on the value of autonomy itself.

The theories agree on one issue: they recognize that a person’s degree of competence is at least relevant for attributing to her the authority to make certain decisions about her own life. On both views a certain point on the scale of competence can be identified at which we are prepared to attribute that authority in a particular case, even if, on the multi-dimensional view, we also have to take other considerations into account in order to do that. How do we identify that point? I will argue that until now only the multi-dimensional theory has been able to provide a plausible answer to that question.^28^ Any plausible suggestion the one-dimensional theory may bring forward in effect introduces an additional relevant dimension.

To the extent they distinguish between the scale and the threshold to begin with, supporters of the one-dimensional view often seem to presuppose that, once the scale has been given, the threshold has been given as well (See e.g. Buller 2001).^29^ But the threshold identifies the level of competence required for ascribing decision-making authority. Therefore the identification will depend on the reasons we have for wanting to ascribe such authority.

The multi-dimensional theory seems to have a simple solution to the identification problem: the more a person has the required cognitive and affective abilities, the more reason we have to trust her decisions.^30^ At the point where we have more reason to rely on her assessment of her relevant interests than on our own, we impute to her the authority to make her own decisions. The right to self-governance, in that case, is to be seen as an indirect realization of the principle of best interests.^31^ But if we accepted that position, it would lead to us deeming ourselves justified to limit a person’s authority in all cases in which we are sure that she is about to damage her own interests. I have mentioned some reasons, such as preserving a relationship based on trust and leaving intact her self-respect, why it is important to respect her wishes, even though we do not scale her competence very high. It follows that the determination of the threshold in the multidimensional theory is the result of a process of weighing pros and cons in which the welfare-interests of the person involved constitute just one element, albeit an important one. It may be true that self-governance on the whole tends to lead to better decisions, as most liberals suppose (including Buchanan and Brock, ch. 1), but even if it doesn’t, self-governance has a value of its own, and it is also the cornerstone of a person’s social status.

If the threshold of ‘sufficient’ competence is seen as the result of a weighing of welfare- and autonomy-interests, the threshold must be variable, since it is obvious that these values are not at stake to the same extent in all cases. On a purely one-dimensional view welfare doesn’t play any role at all.^32^ All that counts is the interest that people have in exercising sovereign control over their own lives and in the recognition of their ‘dignity’. For the effects of their decisions on their own interests they are supposed to have the exclusive responsibility themselves. But if that is true, we should only require people to be able to distinguish between available options and to choose between them, for if they are not, they cannot be said to exercise control. Beyond that there is no reason why their abilities should meet certain threshold requirements at all. The basic objection to the one-dimensional theory is that it cannot account for the actual requirements we make, or anything like them.^33^

Feinberg’s justification of soft paternalism with its appeal to the ‘real’ wishes of the person involved only answers this objection in part, as we have seen. Whether our paternalistic interference is supposed to be ‘soft’ or ‘hard’, it will be received with the same indignation: ‘this is *my* life, it belongs to *me* and to nobody else’. Only when our interference is justified by a false belief which can easily be corrected can we expect that indignation to fade away quickly. The challenge is to prove that the fundamental value which justifies assigning decision-making authority to people is not being furthered when we ascribe that authority to people with abilities below the threshold. That challenge cannot be met if we understand that value as autonomy in the narrow sense of self-governance, for if we do, we cannot deny decision-making authority to any person who values having it and is able to make any decision at all, except in a case like Mill’s.^34^ Any further requirements we make in that case already reflect a weighing of other values against the value of self-governance.^35^

But perhaps the challenge can be met if we understand the value of autonomy in a broader way. In the past few decades many views on autonomy have been developed which differ from one another in many ways, in particular in the role they reserve for the faculty of reason, but at the same time show a reasonable degree of overlap and convergence (Delaere 2010, ch. 3). A human life may be regarded as the life of a particular person because it has a certain internal structure. This structure emerges when that person makes her acts depend on a certain more or less permanent, more or less coherent pattern of values. That she uses this pattern as a guideline is shown by the way she characteristically reacts to the circumstances in which she regularly finds herself and by finding reasons for her choices in certain relatively new constellations of facts. Her personal identity is the result of such choices, taken over at least a certain period of her life. It is this process of shaping one’s own life that we respect by allowing people to take their own decisions, either to their detriment or their advantage.^36^

The notion of ‘shaping one’s life’ is not merely meant to refer to causal influence. You can recognize that the influence of your social environment ‘scaffolding’ your autonomy has been equally or even more important for the actual shape your life has taken than your own exercise of that autonomy (cf. Heath and Anderson 2013), and still rightly consider it your own life, as long as you endorse the result of those influences. Nevertheless, self-governance remains at the core of this broader conception of autonomy, because it is an essential element of the activity of shaping your own life (Glod 2008, 14–15).^37^ At the same time, not every act of self-governance really helps in building or maintaining that shape, and whether we may trust it to do so clearly depends on your decision-making capacities. That is why the allocation of decision-making authority is rightly made dependent on the extent to which you possess those capacities.

But on this approach that cannot be the only relevant factor. In some decisions much more is at stake than in others, not as regards the agent’s welfare, but as regards the authentic character of his future life. It is therefore reasonable to set higher requirements on his competence, the greater the risks for his autonomy in the broad sense, or the lesser the fit between his actual decisions and his underlying enduring values. The resulting conception of the right to self-governance is therefore already a multi-dimensional one.

Recently it has been pointed out that the standard view of competence, as it has been codified by the law in many countries and has been operationalized in the Mac-CAT-T and similar instruments, is defective because, in order to assess a person’s ability to evaluate the consequences of the alternative choices she faces, the values she presently subscribes to are taken for granted. The problem with, for example, anorexia nervosa patients is that they are often quite good in making such evaluations, but that they tend to give inordinate priority to the value of being thin (Tan et al. 2006). Similarly, a depressed patient can very well understand that the choice he is about to make will cause considerable harm to him or even endanger his life; he just doesn’t care (Elliott 1997; Rudnick 2002).^38^ In assessing this criticism of the orthodox conception of competence the basic question is from which point of view the relevant values are being disqualified. Some critics say that these valuations express an underlying pathological condition,^39^ but that explanation is unsatisfactory when it turns out that the condition is considered to be pathological precisely because it expresses itself in non-standard valuations. (If you think being thin is the most important thing in life you must be mad.) This kind of explanation will only work if we have an independent account of pathology.^40^

In many cases, however, another explanation may be available. In such cases the problematic values are not consistently endorsed over time, but rather in very ambivalent and highly variable ways, often for a relatively short time, e.g. a depressive episode. Hence they could be criticized, not for having the wrong etiology, but for being defective in authenticity, out of tune with people’s underlying enduring values (Kleinig 1983, 66–69; Elliott 1997; Rudnick 2002; Charland 2002; Breden and Vollmann 2004; Kluge 2005; Craigie 2011; MacKenzie and Watts 2011; Doorn 2011). Why should we attribute authority to a person’s decisions when she is not satisfied with the priorities expressed in them herself, when she is divided up, unable to speak with one voice? But if that explanation is accepted, it is misleading to consider it as identifying a defect in an additional dimension of competence, ‘valuating capacity’, which until now has not been recognized sufficiently. Competence concerns the ability to process beliefs and priorities in arriving at decisions, but values are a matter of the input of that process. If a defect in valuation is a possible reason for not acknowledging someone’s decision-making authority, that reason has nothing to do with competence, but derives directly from the very point of allocating such authority (on the account of that point we are presently considering). If we refuse to recognize the agent’s authority, it is because of a failure of authenticity, not of any lack of decision-making ability (Edwards 2010).^41^ If competence is the ability to evaluate a proposal in terms of your own stable and coherent set of values, in considering your ability we presuppose that you have such a set. But if you haven’t, that is not itself a defect of competence.^42^

So if we try to justify a one-dimensional account by appealing to the value of autonomy, we end up embracing a multi-dimensional view, even if the considerations it requires us to take into account alongside assessments of competence do not concern welfare interests but authenticity interests. And these sets of interests, though not identical, are not fully independent of each other either. When anorexia patients express their doubts about the absolute priority of being thin, it is because they recognize the danger to other values important to them, including the values of life and health (Tan et al. 2006). These same values are often recognized by depressed patients outside episodes of depression. And this convergence of the two sets of interests, welfare interests and authenticity interests, is not a purely contingent phenomenon. The value of autonomy as I described it, is subjective in the sense that the autonomous person takes the reasons of his choices from his own pattern of values. These values are not subjected to external requirements, e.g. moral ones. But that does not mean that anything that a person identifies as her basic values can be accepted as such, or that all the reasons which she derives from those values must be recognized as her actual ones. Reasons cannot be completely idiosyncratic, they must meet some basic conditions of intelligibility.^43^ If someone agrees that he is in good health, happy on balance in his family-life, his job etc., but nevertheless insists that he wants to die at 42 because Elvis Presley died at that age, we cannot be satisfied with his explanation. There have to be other, more basic reasons. And whether these reasons are self-regarding or other-regarding ones, they have to fit into some intelligible conception of the human good. *Quidquid appetitur appetitur sub specie boni*: everything which people aim at, they aim at because they consider it to be good, though not necessarily only good for themselves. The limits to what they can claim to be aiming at are the limits to what can be so considered. On this basic level respect for autonomy and concern for welfare no longer come apart so easily. Most basically, even in order to recognize a ‘choice’ or ‘decision’ as such, we have to be able to connect it to some possible preferences, i.e. intelligible rankings of the human good.

In considering the possibility of defending a one-dimensional theory of decision-making authority on the basis of a broad conception of autonomy I have explored a line of argument without fully committing myself to it. In particular I have introduced the relevance of doubts regarding a patient’s present values in a hypothetical way only, because a full assessment of this idea would need a more extensive discussion. To begin with, it is obviously dangerous to allow a patient’s decision-making authority to be restricted by appeal to values she presently doesn’t recognize but should recognize. The literature on this issue itself shows how easy it is to slip from an internal to an external criticism of present values, from paternalism to moralism.^44^ The possibility of such slippage compounds the difficulty of establishing a person’s ‘real’ interests, whether welfare or autonomy interests, and our fallibility in this area surely is the strongest argument for a one-dimensional view of the qualifications needed for acquiring the right of self-governance, or at least for adopting it in law. But if other considerations, however suspect, inevitably return in the disguise of concerns about competence, whether it is by introducing the variable standard or by allowing criticism of present values, it is preferable to be open about the paternalistic or even moralistic character of such considerations, if only to be alerted to the extra caution required for allowing them any weight.

Of course I have not proven that an explanation of the sufficiency of ‘sufficient’ competence cannot be given by supporters of a one-dimensional account. My aim has only been to challenge them to provide it. As long as they haven’t we have good reason to prefer a multi-dimensional theory.

## Conclusion

In general competence in the binary sense is taken to be a ‘gatekeeping’ concept (Faden and Beauchamp 1986, 287 ff). Our assessment of competence, e.g. in a medical context, allows us to sort patients into two categories: those from whom we have to obtain informed consent before proceeding to treat them, and those whose interests are not entrusted to their own care, but to the care of someone else (either the doctor or a legal representative). The gatekeeping nature of that assessment suggests that the procedure for decision-making about treatment consists of two steps, even if the second step can sometimes be made redundant by the first. The first step establishes whether or not we have to request the consent of the patient. If we have to, and the patient refuses, we can still decide whether or not to abide by this refusal: that is the second step. It is a substantial normative position, by now seemingly supported by the law in most countries, that we should always abide by ‘competent’ (i.e. sufficiently competent) refusals.

If the variable standard is introduced, this two-step model is seemingly kept intact. Even the prohibition against ‘hard’ paternalism is formally maintained (e.g. Berghmans 2000), but that is no longer a substantial normative position, but, as I explained, a mere tautology. In fact, all considerations which could be relevant in the second step, already count in making the first. The fact that we keep thinking in terms of the two-step model after having actually abandoned it, leads to all sorts of conceptual confusion. On the one hand we are often insufficiently aware that we make judgements of (binary) competence which are not only informed by assessments of decision-making abilities, but by a number of other considerations as well. On the other hand we tend to be insufficiently aware that we only manage to avoid ‘hard’ paternalistic policies because we have already considered all reasons for such policies in deciding about ‘competence’.^45^

To end these confusions the best we could do is to stop using the concept of competence in the binary sense altogether. When we claim that someone is ‘sufficiently’ competent to be allowed to take a certain decision even though that decision is a mistaken or suboptimal one, we already admit that there are other relevant considerations except (scalar) competence, but that they are insufficient to deny the person’s authority in this case. We then actually say something not only about the competence of the person involved, but also about the balance of all relevant considerations. It would then be in the interests of clarity to focus on the issue of a person’s decision-making authority directly, and in discussing it distinguish between considerations concerning ‘competence’, i.e. decision-making abilities, risks of harm, the centrality of decisions to the agent’s sense of self, the invasiveness of interventions etc.

As a consequence we should also give up the distinction between hard and soft paternalism. In all cases the same considerations, including those regarding a person’s decision-making abilities, are relevant for making the one and only decision we have to make on the basis of them: whether or not to respect a person’s unwise decision.^46^

In “The distinction between hard and soft” section I have argued that we have moral reasons to avoid soft paternalism, by respecting a supposedly incompetent person’s wishes as much as possible. In an important paper Daniel Groll agrees, but insists hat there is still a fundamental difference between those reasons and the reasons for respecting the wishes of a competent person. If we don’t want to upset someone by disregarding her wishes or get into unnecessary conflict with her, we consider the satisfaction of those wishes only as an element of her wellbeing, we do not really attribute any authority to them (Groll 2012). But that is only true of some of the reasons I mentioned in that section, not of the fundamental ones. If, for example, we want to avoid shattering an Alzheimer patient’s self-esteem as much as possible, the basic reason need not be that by doing so we will make her unhappy, but that we fail to respect her as an agent with some remnants of autonomy and as a member of the moral community in her own right, albeit perhaps no longer a fully equal member. We may therefore allow her to make some mistakes that are self-harming, even self-harming on balance, when feelings of frustration and loss of trust have been factored in.^47^ But in that case we really attribute to her a kind of authority . And when we limit that authority because the harmful effects of her exercise of it are too great, our paternalism is justified, if it is, by balancing autonomy- and welfare-interests, not because it is of a special harmless kind, identifiable as ‘soft’ paternalism.

It may be true that the two-step model is already too much entrenched in the language of the law to give my revisionistic proposal any chance of success (Cf. Buchanan and Brock 1990, 67). On the other hand, the one-step multi-dimensional model of making such decisions seems to be much closer to the way they are actually made in practice.^48^

## Footnotes

1 Cf. the abilities measured by the internationally best-known of the many existing instruments for the assessment of decision-making ability in a medical context, the MacArthur Competence Assessment Tool or MacCAT-T (Grisso and Applebaum 1998). It is controversial whether the ability to deliberate is an essential component, see below. The inclusion of the capacity to communicate one's decisions to others is in any case mistaken: an aphatic and paralyzed patient or a patient in a locked-in-syndrome can be fully competent, even if it is hard or impossible to establish this. Interestingly, the MacCAT-T only provides a scale, not a cutoff point.

2 Occasionally ‘competence’ is used for the scalar meaning, ‘capacity’ for the bivalent one, or vice versa. Or one of the two is said to be a legal term (Ganzini et al. 2005).

3 De Marco (2002), discussing an example of Wilks (1997), argues that if two tightrope walkers are equally competent and we refuse one of them to work because he doesn't intend to use a safety net, the refusal cannot be explained by his lack of competence [Competence being a ‘genuine attribute of persons’, (Culver and Gert 1990, 635; Checkland 2001, 37)]. But that the tightrope walkers are equally competent in the scalar sense clearly doesn't show that both of them are sufficiently competent for executing different tasks. And whether the tasks of these tightropers can be differentiated by the different risks they are prepared to take is the open normative question in dispute.

4 Cf. the definition of incompetence in the US Mental Capacity Act (Department of Constitutional Affairs 2005, section 3), or in the relevant laws of some Canadian provinces as quoted by Kluge (2005).

5 Why is competence a task-related concept? A possible explanation is that different tasks imply different levels of risk. Authors opposing the variable standard of competence owe us an alternative explanation.

6 Following Buchanan and Brock (1990, ch. 1), this is usually called the 'process' view. But that term suggests a mental process in real time, which need not occur.

7 In the MacCat-T understanding is supposed to be a general capacity, but appreciation is taken to be the ability to grasp the significance of the acquired information to one’s own case.

8 The patient may, for example, be requested to participate in research, but attribute therapeutic intent to the proposal (Lidz and Appelbaum 2002). For the same reason tests of competence which use vignettes (hypothetical treatment decisions) are unsatisfactory (Welie 2001, 144).

9 It could be objected that in such cases of ‘thinking without thinking’ the actual thinking has been done on prior occasions. But according to many social psychologists experts are able to make spontaneous judgments concerning problems they never have met before (see e.g. Dijksterhuis 2004). In any case for an assessment of competence it is normally impossible to check whether any prior thinking has occurred.

10 The importance of emotional factors has been stressed and explained by Elliott (1997), Charland (1998a, b), Wijsbek (2000). Culver and Gert (1990) purely focus on cognitive abilities because they don't count the ability to evaluate consequences as a relevant one. But why should we recognize the decisions of a person who lacks that ability to have any authority? That would be like giving someone the authority of a judge who is good enough in establishing the facts of a case, but unable to apply the law to them.

11 Suggested by Roth et al. (1977), proposed by Drane (1985), explained and defended by Buchanan and Brock (1990, ch. 1), and by Berghmans (2001), accepted by Beauchamp and Childress (2009, 116–117). It is reported to be universally subscribed to in Dutch health care practice (Gevers and Dute 2011). For Feinberg’s view see footnote 22.

12 It has subsequently been discussed in various other contributions to the same journal. Most of these contributions do not sufficiently distinguish between competence and authority and/or competence in the scalar and threshold sense. The argument had already been used in criticizing Buchanan and Brock (1990) by Culver and Gert (1990, 636–637).

13 Of course, in many cases the right decision can be a matter of reasonable debate, but even in most of these cases some choices could be made that would raise suspicions of incompetence.

14 The term has been introduced by Hart (1958) see Hart (1982, 18, 254), but the idea can be found as early as Hobbes’ *Leviathan*, ch. 25; cf. den Hartogh (2002, ch. 6). For reasons I have explained in den Hartogh (2002, ch. 7). I reject the supplementary analysis of authority in terms of ‘exclusionary’ or ‘peremptory’ reasons, proposed by Joseph Raz in many works since Raz (1975), and accepted by Hart, o.c., and by Groll (2012), among many others.

15 Unless by changing his mind he can be taken to indicate that his competence in the scalar sense has improved, and this indication can be independently confirmed.

16 Wilks (1997), footnote 20, considers this position, and proceeds to provide a counter-example, but that is nicely dealt with by Wicclair (1999), see also Checkland (2001).

17 Particularly in the case of the state’s authority; cf. den Hartogh (2001).

18 This is an example about coordinative rather than epistemic authority. For the distinction see den Hartogh (2002, § 6.2), with references. The point that authority is normally only content-independent within limits applies to both types of authority.

19 If the risks are greater, it may be more difficult to understand and evaluate the options (Wicclair 1999). But it may also be more obvious what one should do (Buller 2001, 100–101). In both cases requiring a higher or lower level of competence doesn’t amount to using a variable standard, because the risks are not considered to be relevant in themselves.

20 Two authors have been credited with introducing the distinction: Dworkin (1972) and Feinberg (1971).

21 “I do not like to be told what to do. It is my body, I have my own opinions and I speak my own mind. If they want something from me, I just say ‘no’.” Says the demented mrs. Jaspers as described by Schermer (2002, 116, 126). According to Feinberg (1986, 12–16), a person's insufficiently voluntary choices are completely foreign to him. Arneson (2005, 6), points out that this is an exaggeration, and that it puts a burden on the determination of the threshold of ‘sufficient’ voluntariness which it cannot bear.

22 At least as far as the criminal law is concerned. Feinberg (1986, 117–20), however, proposes a sliding-scale test of voluntariness, in the interest of autonomy only, in order to minimize the consequences of mistakes. But Buchanan and Brock (1990, 44–46) rightly point out that higher standards may well decrease the risk that an insufficiently voluntary choice is given undue authority (false positive), but at the same time increase the risk that a sufficiently voluntary choice is overridden (false negative), which should be more worrisome if it is only autonomy which counts (Cf. Breden and Vollmann 2004, 279). Hence Feinberg's proposal cannot be understood, unless he is already balancing autonomy- and welfare-interests.

23 For the same reason it would not make sense for adherents of the variable standard to claim that false negatives are more important in determining the threshold of competence than false positives or v.v. (see footnote 22). For we can only judge a person sufficiently competent for his decision to be authoritative by weighing those dangers.

24 Cf. Griffin (2008, 67 ff.): the value of human rights admits of degrees, because infringements on them can be attacks on something nearer or further from the centre of one’s normative agency.

25 In the United States, the UK, the Netherlands and most other jurisdictions (but not for example in Scotland or New South Wales), a mentally ill person who is a risk to himself may be forcibly hospitalized and/or treated, whether or not he is deemed incompetent.

26 Such authors might also accept the relevance of the additional considerations for assessing paternalism, hard or soft, which I mentioned in the foregoing paragraph.

27 As has often been observed, particularly a utilitarian such as Mill has great difficulty answering that objection.

28 Or to the related question mentioned in footnote 5.

29 Or see footnote 3.

30 This applies not only to his own best interest in the strict sense but also to effectively achieving his priorities, including those aimed at other people’s interests or the fulfillment of impersonal values.

31 On this interpretation the multi-dimensional theory would not recognize a plurality of relevant values.

32 I.e. in identifying the threshold. Of course, on Mill’s utilitarian view it is suggested, however implausibly, that, by disregarding welfare considerations on this point, ultimately welfare is best promoted.

33 Groll (2012), recognizes, on the one hand, that competent people often make poor choices, and, on the other, that some supposedly incompetent people, still have a ‘will’, i.e. a picture of how their life should go and some ability to make choices accordingly. But, nevertheless, he claims that competent people are distinguished because they have an ‘intact’ or ‘properly functioning will’. (Footnote 46) That is a deeply mysterious notion, in particular when you realise (as Groll does) that competence is a task-related concept. And Groll makes no attempt to explain it.

34 Perhaps one could object that such a weak standard of competence is insufficient to warrant concluding that the person is accountable for his exercise of authority (Wicclair 1991; Elliott 1991). But the challenge remains to determine an alternative standard in terms of this notion without in the process sliding into a multi-dimensional theory.

35 Cf. Arneson (2005, 7), on the enormous importance in a theory like Feinberg's of the line between self-harming choice that is not quite voluntary enough and choice that just passes the threshold. This criticism depends for its cogency on the absence of any principled reason for drawing the demarcation line at some particular place. Otherwise it would be merely an example of the fallacy of supposing that a vague borderline isn't a borderline at all.

36 It is not necessary to regard the creation of a personal identity itself as the fundamental human project. It could be valuable even if it is only a byproduct of a person’s enduring orientation towards other values.

37 He goes on to argue that, though this is a strong consideration against paternalism, it is not necessarily a decisive one.

38 See also Charland (2002) on addiction and competence, and the case of a personality disorder described by Winburn and Mullen (2008).

39 Cf. Christman (2009), arguing that even a full endorsement of one’s preferences on reflection is not sufficient for being considered autonomous if one’s acquisition of those values is due to suspect causal influences.

40 In saying this I do not mean to dispute that non-standard valuation can be an indication of pathology, cf. footnote 42, though the procedure has its dangers, as I point out at the end of this section.

41 It is sometimes argued that distorted value systems lead to emotional blockades in the proper weighing of evidence, and hence are relevant for assessing competence. But the weighing is only considered improper because the value systems are deemed distorted. For a subtle example see Halpern (2010): the evidence her patient cannot weigh properly concerns her own stable priority ranking when the emotional effects of her recent traumatic experiences have subsided. Cf. also Grisso and Appelbaum (2006).

42 As e.g. Charland (2002) supposes. Similarly compulsion is not itself a defect of competence, though observing it pre-empts assessments of competence.

43 This has been persuasively argued in a number of articles by Philippa Foot, collected in Foot (1978), and previously by Anscombe (1957, 70f). Her famous example is a person who says he wants a saucer of mud but is unable to explain what for. Cf. Harry Frankfurt’s comments on Hume’s statement that “Tis not contrary to reason to prefer the destruction of the whole world to the scratching of my finger”, (Frankfurt 2006, 29ff).

44 To mention some examples: Kluge (2005, 298), criticizing a patient’s decision to refuse life-saving treatment in order not to become a burden on her family as showing a lack of self-respect. Arneson (2005, 276–83), arguing for the justifiability of paternalism by pointing to the duty to make something worthwhile of one’s life. Jansen and Sulmasy (2002), arguing for strong restrictions on the permissibility of palliative sedation by appeal to patients’ supposed enduring ‘interests’ in retaining consciousness at all times. I do not deny the validity of such moralistic considerations as such, though none of these authors show how they justify restricting an agent’s liberty. For similar concerns see Faden and Beauchamp (1986, 262–9), Buchanan and Brock (1990, 35–36, 56–57), van Willigenburg (2000).

45 "We believe that open recognition of the limited but important exceptions to the principle of never overruling a competent patient's refusal would better preserve the freedom of competent patients than the present hypocritical acceptance of the absoluteness of the principle" (Culver and Gert 1990, 622).

46 One could still speak of ‘hard’ paternalism in any case of paternalistic intervention in the execution of decisions which the agent had the authority to make. But then hard paternalism would be wrong by definition. Even as for justified paternalism, in some cases it could be correctly characterised as being ‘harder’ than in others.

47 According to Groll in that case we are still not really recognizing her authority, because we respect her as a decision-making agent only to the extent that we consider it part of her well-being to be so respected. But I can’t see why it matters whether we have such an ulterior reason. Moreover, we need not have that reason in the case of supposedly incompetent agents, and might have it in the case of the supposedly competent ones.

48 Cf. the description of the case of Mrs. Jaspers in Schermer (2002, ch. 7) and her conclusion, 132-3.
